# Microwave Discharges in Liquid Hydrocarbons: Physical and Chemical Characterization

**DOI:** 10.3390/polym13111678

**Published:** 2021-05-21

**Authors:** Yuri A. Lebedev

**Affiliations:** A.V. Topchiev Institute of Petrochemical Synthesis of the Russian Academy of Sciences (TIPS RAS), Leninsky Ave. 29, 119991 Moscow, Russia; lebedev@ips.ac.ru

**Keywords:** microwave discharge, discharges in liquids, microwave discharge in liquid hydrocarbons, methods of generation, plasma properties, gas products, solid products, plasma diagnostics, plasma modeling

## Abstract

Microwave discharges in dielectric liquids are a relatively new area of plasma physics and plasma application. This review cumulates results on microwave discharges in wide classes of liquid hydrocarbons (alkanes, cyclic and aromatic hydrocarbons). Methods of microwave plasma generation, composition of gas products and characteristics of solid carbonaceous products are described. Physical and chemical characteristics of discharge are analyzed on the basis of plasma diagnostics and 0D, 1D and 2D simulation.

## 1. Introduction

Recently, electrical discharges in liquids [1,2,3,4,5,6,7,8,9,10] and, in particular, microwave discharges [11,12,13] have been intensively studied. Microwave discharges in liquids are less studied. These discharges exhibit properties that distinguish them from widely used DC, HF and high-voltage discharges. They can be used to produce hydrogen, coatings, nanoparticles and nanotubes, for water purification, etc. [13]. Several papers on modeling of electrodynamics and plasma processes in such discharges have been published [14,15,16,17,18,19,20,21,22,23,24,25,26]. Microwave plasma in liquids is an extremely interesting object for investigation, since it is often non-equilibrium, heterogeneous, with large spatial gradients of parameters. Plasma as a rule is unsteady and existing under conditions of permanent exchange by energy and particles with the surrounding liquid medium.

One of the key problems of discharges in liquids is answering the question of what is first: discharge produces the gas bubble or discharge is generated in the existing gas bubble. There are several theories of bubble generation by discharges, which are reviewed in [6,10]. Considering the known results on microwave discharges, it can be stated that, although they talk about microwave discharges in a liquid, in fact microwave plasma is created in a gas bubble (a few millimeters in diameter) inside the liquid. The bubbles can be created in different ways: (a) by evaporation of the liquid in the vicinity of the antenna tip heated by the microwave field; (b) by creation of artificial bubbling by introduce of additional gases (argon is most often used); and (c) by action of ultrasonic waves. The surface of the bubbles is located near the high-temperature plasma zone, which ensures a high-rate input of evaporated molecules of fluids in the bubble. This bubble can be considered as a mini plasma chemical reactor. As a result of the intense flow of molecules into the bubble, high concentrations of active particles (atoms, radicals and charged particles) are created in the plasma. Therefore, the efficiency of physical and chemical processes in this reactor is high. Accordingly, the rate of formation of products is also high. Moreover, the small dimension of plasma region produces high gradients of parameters and high rate of stabilization of products. These features are the major differences from conventional gas phase microwave plasma processing.

This article summarizes the results obtained in the study of microwave discharges in liquid hydrocarbons. Almost the entire spectrum of liquid hydrocarbons has been used in studies of microwave discharges in liquids including alkanes (C_n_H_2n+2_), cyclic (C_n_H_2n_) and aromatic hydrocarbons, industrial and engine oils and their wastes, oil and heavy oil products.

Since gas and solid products are formed in such discharges, as well as the liquid itself is exposed to the discharge, all these results are consistently considered in the article. In addition, the results obtained in the diagnosis and modeling of such discharges are described.

It should be noted that the dielectric constant is approximately the same for non-polar liquid hydrocarbons (ε ~ 2.0) and the tangent of losses for non-polar hydrocarbons is of the order of 10^−4^, thus the loss of microwave energy for heating the liquid is small. Unfortunately, in the process of burning a discharge in liquid hydrocarbons, one of the products is a solid carbon-containing phase, which naturally increases the loss tangent. For polar liquids, the loss tangent is greater, and the dielectric parameters depend on temperature.

Note that non-polar hydrocarbons are transparent in the visible wavelength range; this is important for carrying out spectral-optical measurements, which are practically the only method for diagnosing such discharges. In the process of burning a discharge in hydrocarbons, solid particles appearing in a liquid reduce its transparency.

## 2. Experimental Setups and Methods of Diagnostics

**Figure 1:**
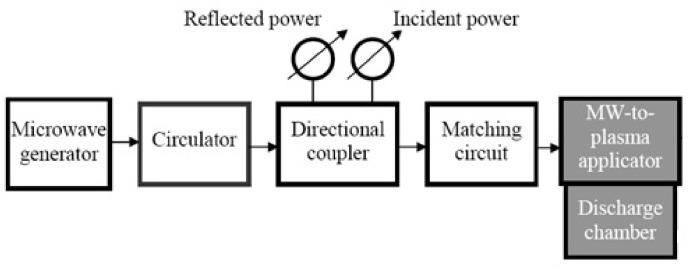
The general scheme of microwave discharges setup.

The general scheme of microwave discharges setup in liquids does not differ from that used to generate microwave discharges in the gas phase (Figure 1). It is considered in detail in [27] and includes a microwave generator (as a rule, magnetrons with a frequency of 2.45 GHz and a power of up to several kilowatts in continuous or pulsed modes), a circulator to protect the generator from reflected power, an incident and reflected power meters, matching device, discharge chamber, gas supply system, exhaust gas sampling and exhaust pump system. Discharges in liquids are created at pressures from units of kPa to atmospheric pressure. The pressure in the plasma region is determined by the pressure provided by the pumping system above the liquid surface and differs from it by the pressure of the liquid column above the plasma region.

**Figure 2:**
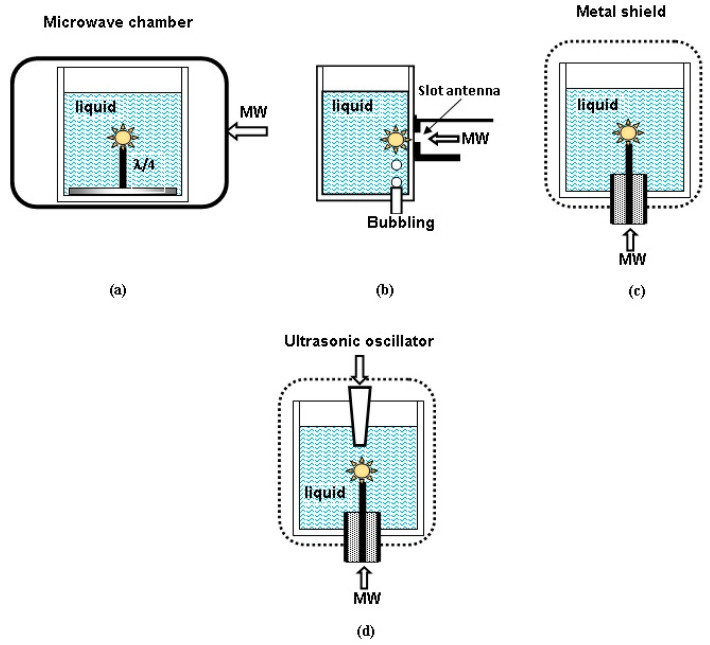
Schematics of different types of microwave discharges in liquids: (**a**) discharge on the base of quarter-wavelength antenna; (**b**) discharge on the base of slot antenna; (**c**) discharge with energy input through the coaxial line; and (**d**) microwave discharge in combination with ultrasonic radiation.

Various microwave-to-plasma applicators are used for generation of microwave discharges in different liquid dielectrics. Their task is to create the microwave field, having the strength sufficient to create and sustain a discharge in the gas bubble. There are four main types of discharge devices (Figure 2). Plasma is generated near microwave antennas of different types.

The first and simplest one is the use of quarter-wavelength metal antennas mounted on the metal basement (Figure 2a), at the end of which a discharge is ignited in the gas bubble. The antenna length should be equal to a quarter of the wavelength of the liquid radiation ($\lambda =c/f\sqrt{\varepsilon }$, where *c* is the speed of light in vacuum, *f* is the frequency and *ε* is the dielectric constant of the liquid). For non-polar liquid hydrocarbons, the antenna length is about 2 cm. This means that the antenna length is practically the same for all such hydrocarbons. The antenna is placed in a heat-resistant glass with low loss in the microwave frequency range, filled with liquid hydrocarbon. The glass is located in the microwave chamber. Therefore, energy can be supplied to the antenna through the liquid layer. Since the strength of the microwave field at the antenna increases with a decrease in its diameter, the power required to initiate a discharge decreases. Additional gas can be supplied via a channel in the antenna.

This method is implemented, for example, in [28,29,30,31]. A conventional microwave oven was used as a microwave chamber [28], and the discharge was ignited in a glass placed in the oven chamber. To increase the efficiency of plasma processes, not one antenna can be used, but a system of whip antennas [28]. A special metal chamber into which the microwave energy was supplied from three magnetrons is described in [29,30,31].

One of the disadvantages of this system is the destruction of the end of the antenna in the plasma or the growth of deposits on it. This leads to a change in its length and violation of the condition of its equality to a quarter of the wavelength and to unstable operation of the device. To reduce the role of antenna erosion, various methods are proposed, in particular the use of refractory metals (such as molybdenum or tungsten), coating of antennas with heat-resistant dielectrics (see, e.g., [14]) or the use of pulse regime of microwave generator [32]. Antennas of various shapes are used to excite the discharge. For example, the authors of [33] described the use of a broken ring antenna.

The second type of antenna used is the slotted antenna (Figure 2b). Gas bubbles are created near it surface due to heating of liquid by microwaves (it should be noted that many bubbles can appear simultaneously along the slot) or by the introduction of additional gas (the so-called “multibubble system”) [34,35,36]. Bubble formation at the antenna leads to unstable operation of the device and a decrease in the efficiency of energy transfer to the discharge. Simulations and experiments have shown that this can be eliminated by introducing a bubble control element, which is a quartz plate with holes placed close to the antenna [35]. Multiple slot antennas can be used to increase the efficiency of the device. By artificially creating bubbles, plasma can be created at pressures up to atmospheric pressure.

Another method of microwave plasma generation in liquids is the coaxial input of microwave energy into the liquid, in which the discharge is ignited at the end of the central conductor of coaxial line (Figure 2c). It is the most widespread method of microwave plasma generation in liquids (see, e.g., [14,15,28,37,38,39,40,41,42,43,44,45]). Modeling of electrodynamics of quarter wavelength [30] and coaxial [44] antenna systems has shown that in the latter system the microwave field strength at the tip of antenna is much less sensitive to the antenna length. As plasma is generated at the tip of antenna, this provides more stable operation of plasma generator.

All problems with antenna erosion considered above exist also in this case. The methods of suppressing this effect are also the same.

A more complex method can be used, in which both the microwave and ultrasonic radiation simultaneously act on the liquid (Figure 2d) [46,47,48]. In this case, acoustic oscillations (with a frequency of 24.5 kHz, 10–30 W) are a source of bubbles in a liquid, in which a plasma is created when exposed to microwave radiation (2.45 GHz, 50–200 W). The authors of [46] called it “sonoplasma”, and, in contrast to “sonoluminescence,” it gives a constant radiation in time, which is caused by the absorption of microwave energy and not by the energy released during bubble collapse. After ignition of the discharge, ultrasonic radiation can be switched off and discharge maintained only by microwave radiation.

Discharges in liquids are generated at both reduced pressures and atmospheric pressure. Generally, microwave generators with a frequency of 2.45 GHz are used to create the plasma.

Note that, if one does not take special measures, microwave discharges in carbon-containing liquids are nonstationary [43]. This is due to the formation of solid carbon-containing particles, which are distributed in the volume of the liquid due to convective flows. This leads to the absorption of microwave radiation, decreased liquid transparency for plasma radiation and discharge failure. To eliminate this phenomenon, it is necessary to organize the circulation of the liquid with its purification from solid particles. This must be done when creating technological processes.

The appearance of carbon-containing solid particles in the liquid is also manifested in another effect [43]. It leads to the fact that the composition of the liquid wall evaporating into the bubble contains more carbon than at the beginning of the experiment in pure hydrocarbon and the discharge is maintained in a medium with a time-varying composition of the evaporating boundary. Thus, the nonstationarity of the discharge in liquid hydrocarbons is associated not only with the formation of a gas bubble with plasma and its dynamics, but also with a change in the composition of the evaporating liquid wall of the gas bubble during the course of the discharge burning and the associated change in the composition of the gas phase in the bubble.

Microwave discharges in liquids are extremely difficult for experimental study. Using contact diagnostic methods is difficult because the plasma is in a microwave field and the insertion of foreign objects in plasma distorts both the structure of the microwave field and plasma. In addition, the microwave field can lead to the damage of diagnostic devices. Therefore, the only method for studying the plasma parameters is the optical methods. Most papers contain results of optical emission spectroscopy (these results are mainly related with measurements of excitation temperatures of plasma particles and gas temperatures).

As discharges in hydrocarbons produces the gas and solid particles, to study these components, various special methods of diagnostics are used: gas chromatography, mass-spectrometry, Raman spectroscopy, field emission scanning electron microscope, etc.

Additional possibilities in study of discharge parameters are provided by modeling, which is rather complicated, since the discharge is non-stationary and constantly exchanges in energy and matter with the liquid wall. The results of zero-, one- and two-dimensional modeling of processes in plasma are described in [14,15,16,17,18,19,20,21,22,23,24,25,26].

## 3. Gas Products of Microwave Discharges in Liquid Hydrocarbons

**Figure 3:**
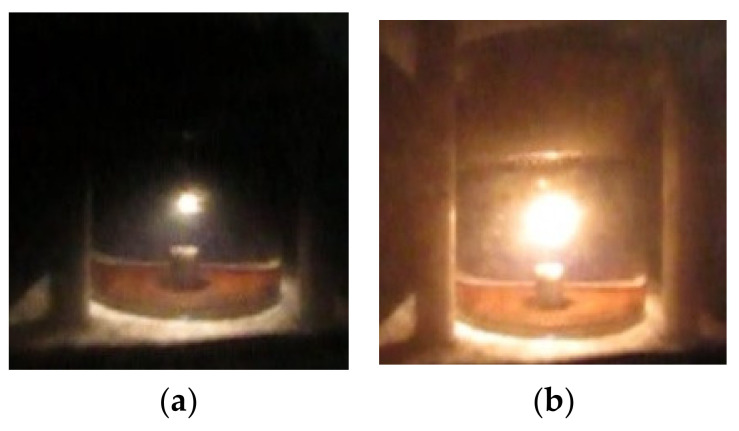
Two successive frames of microwave discharge in engine oil obtained in the installation with quarter wavelength antenna described in [29,30,31] (shooting speed is 240 frames per second, time interval between figures (**a**) and (**b**) is of 4 ms).

**Figure 4:**
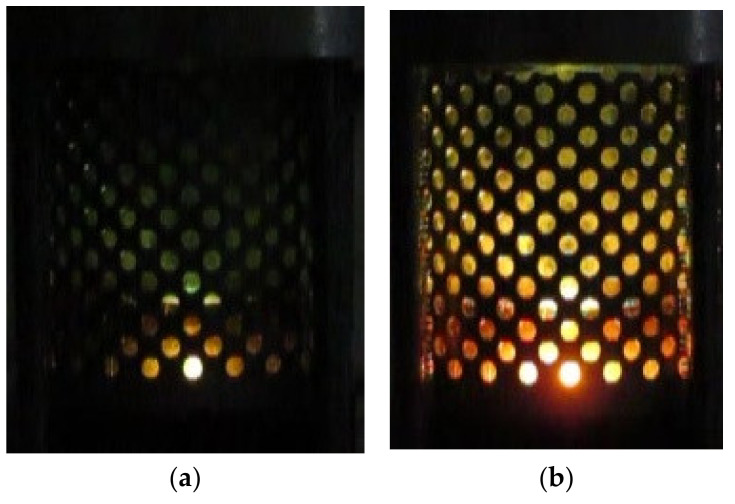
Two successive frames of microwave discharge in engine oil in installation with coaxial input of microwave energy described in [41,43,44,45] (shooting speed is 240 frames per second, time interval between figures (**a**) and (**b**) is of 4 ms).

**Figure 5:**
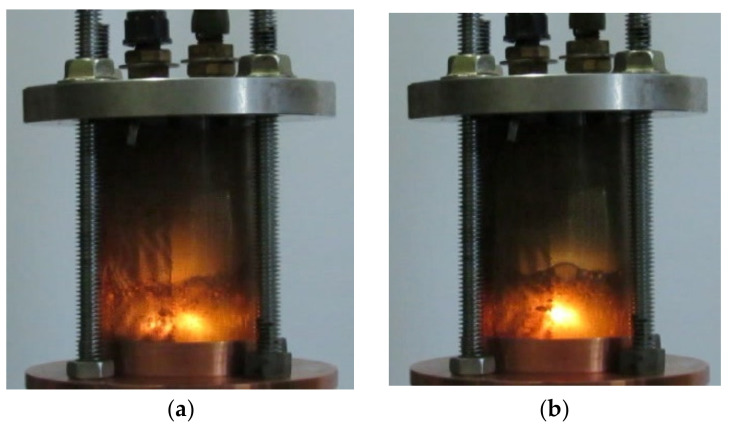
Two successive frames of microwave discharge in *n*-heptane in installation with coaxial input of microwave energy described in [41,43,44,45] (shooting speed is 25 frames per second, time interval between figures (**a**) and (**b**) is 40 ms).

As noted above, almost the entire spectrum of liquid hydrocarbons has been used for studying microwave discharges in liquids. For example, Figure 3 shows two successive frames of microwave discharge in the engine oil obtained in the installation with quarter wavelength antenna described in [29,30,31]. The figure shows that discharge exists at the tip of antenna. Two successive frames of microwave discharge in the engine oil obtained in installation with coaxial input of microwave energy described in [41,43,44,45] are shown in Figure 4. Figure 5 shows the frames of discharge in *n*-heptane in the same installation. The presented photographs reflect the non-stationary nature of microwave discharges in liquids.

Gas products of microwave discharge in *n*-heptane are studied much better than in other hydrocarbons [43], and it is possible to compare these results with the results obtained in other conditions.

Since *n*-heptane is the main component of reference and surrogate fuels for internal combustion engines, and is a convenient object for studying thermal transformations of higher *n*-alkanes, much attention is paid to the study of its combustion and oxidation processes [49,50,51,52], as well as pyrolysis [53,54,55]. Data on the composition of the products and the kinetics of pyrolysis of *n*-heptane were used to establish the mechanism of its oxidation and combustion and to build the kinetic schemes of processes [56].

It is of interest to compare the results on the gas-phase products of a microwave discharge in liquid *n*-heptane with known data on the decomposition of *n*-heptane under various conditions.

The results of experimental study of pyrolysis of *n*-heptane are partly summarized in the reviews [53,54,55]. Processes were studied in a flow reactor when the reagent was diluted with nitrogen [57,58], argon [59,60], water vapor [61,62] and without dilution with a carrier gas [63], as well as processes in the shock tube [64,65]. It was found that decomposition of *n*-heptane under the action of temperature is a radical-chain process and is described by the Kosyakov–Rice mechanism with additions [53,54,55,57]. The main products of pyrolysis of *n*-heptane are hydrogen, methane, ethylene, ethane and propylene [57,58,59,60,61,62,63]. α-olefins C_4_–C_6_ (butene-1, pentene-1 and hexene-1) are also always present in the product mixture; the first of them at moderate temperatures and small degrees of conversion belongs to the main products. Among the products, the following are also identified: butenes-2, butadiene-1,3 and pentenes. In minor and trace amounts at pressures of the order of 0.1 MPa and below, there are propane, *n*-butane and *n*-pentane. As the pressure increases, the rate constant of *n*-heptane decomposition increases and the selectivity of products changes due to the faster growth of the rates of bimolecular processes, such as the detachment of the H atom leading to the formation of alkanes, as compared to monomolecular reactions such as the breakdown of radicals, leading to the formation of ethylene [53]. As a result, the selectivity of the formation of hydrogen and ethylene decreases, while the formation of *n*-alkanes, starting from ethane and α-olefins increases [59,63]. Increase in the temperature leads to increase in the selectivity of the formation of light products, especially methane, ethylene and hydrogen [53,63]. With a further increase in temperature to 1300 K or more, new products appear, such as acetylene, methyl acetylene, allene, vinyl acetylene, pentadiene-1,3 isomers, hexadiene-1,5 and benzene [60,64,65].

Decomposition of *n*-heptane was also investigated in a barrier discharge [66,67,68]. It is established in [66] that monotonous growth of the methane, ethylene, ethane and propane yields is observed with increase in the electric field strength. In [67], the influence of the alkane structure and the type of diluent gas on the yields of hydrogen and other products is investigated. Gas phase products were determined for *n*-heptane: hydrogen, methane, hydrocarbons C_2_ (ethylene + acetylene, ethane), C_3_ (propylene, etc.) and C_4_. In [68], a kinetic study of the conversion of *n*-alkanes C_6_–C_9_ is carried out. It is established in [67,68] that, as the number of C atoms in the alkane molecule increases, its degree of conversion and energy efficiency increase, in contrast to the results obtained in [66].

Comparison of distributions of gas-phase products in pyrolysis of *n*-heptane in different conditions is given in Table 1.

In microwave discharge [43], among the products, acetylene and its homologs, as well as allene, absent in low-temperature experiments [57,59,61,62], were determined. The same products were fixed at temperatures of more than 800 °C in flow reactors [58,60] and in the shock tube [41,42]. High yields of acetylene and hydrogen are obtained in [43], which indicates high temperatures in the discharge. This is confirmed by estimated temperatures based on the emission Swan bands.

Comparison of the results of chemical analysis of products obtained in a microwave discharge [43] in liquid *n*-heptane and barrier discharge [66,67,68] showed that the composition of products obtained in microwave discharge is much richer.

The main gas products and their related concentrations in microwave discharge in *n*-heptane are in agreement with the results of modeling [22,25,26]. Composition of main gas products of microwave discharge in different hydrocarbons is shown in Table 2.

The results shown in Table 2 can be summarized as follows:

- Increase of the molecular weight of alkanes is accompanied by increase in the yield of acetylene and decrease in the yield of hydrogen (in the series C_7_–C_16_, including cycloalkanes).
- Hydrogen and acetylene are predominantly formed in aromatic compounds.
- Gas products of microwave discharge in the investigated liquid cycloalkanes and aromatic compounds without radical groups practically do not contain methane or ethylene. As the number of radical groups increases, the composition approaches the composition of the discharge products in alkanes.

Estimations based on results of experiments with microwave discharge in liquid dodecane showed that the amount of energy needed to generate 1 mol of hydrogen is 640 kJ/mol [28].

Hydrogen is the main component of exhaust gases of microwave discharge in any liquid hydrocarbons independently of the method of plasma generation. This is illustrated by the results in [48], where microwave discharge is generated in the presence of ultrasonic oscillation (Figure 2d) at pressure 250 hPa. Measured volume concentration of hydrogen in different liquids were: 81/79 in *n*-dodecane, 67/76 in benzene, 66/73 in cooking oil, 71/65 in waste cooking oil, 74/65 in engine oil and 73/65 in waste engine oil (here, the numerator corresponds to the results obtained in case of combine action of microwave and ultrasonic radiation and the denominator corresponds to the results obtained in the case of switching off the ultrasonic radiation just after the ignition of discharge).

**Figure 6:**
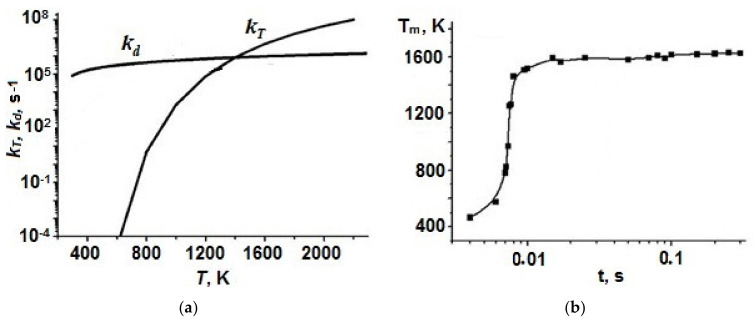
(**a**) Rate constants of thermal dissociation (*k_T_*) and electron impact dissociation (*k_d_*) at microwave field 1.5 × 10^4^ V/cm and electron density n_e_ = 10^14^ cm^−3^; and (**b**) change of maximum gas temperature in time.

Several comments should be done on the role of electron impact in the process of hydrocarbon decomposition in microwave discharge in liquid hydrocarbons. This problem was considered in detail for the case of plasma in liquid *n*-heptane [19,20,69]. 2D modeling shows that:

(i) Electron-impact dissociation occurs only in a narrow region adjacent to the electrode because the microwave field is concentrated near the end of central electrode and falls rapidly with increasing distance from it.
(ii) Electron impact affects the dissociation of *n*-heptane only during a short time period from discharge ignition (≤10^–3^ s), when the gas temperature is low (<1300 K).
(i) At times greater than 10^–3^ s, the dissociation of *n*-heptane occurs thermally. The role of plasma electrons in the decomposition of *n*-heptane is reduced to heating the gas inside the plasma bubble (Figure 6).

## 4. Solid Products of Microwave Discharges in Liquid Hydrocarbons

It is known that MW discharges in liquid hydrocarbons are accompanied by formation of nanosize carbon-containing particles (see, e.g., [28,31]) and the liquid becomes saturated with black particles and loses its transparency both for microwaves and light.

The first question is where the solid phase is formed by the discharge in liquid hydrocarbons, namely in the liquid phase or in the region of gas-phase microwave discharge. On the one side, numerous investigations of various gas discharges in carbon-containing media at atmospheric pressure point to efficient formation of sooty particles. On the other side, in the case of in-liquid microwave discharge, the question is caused by the fact that insignificant amounts (<1%) of polycyclic aromatic hydrocarbons are detected in liquid hydrocarbon after centrifugation and isolation of dispersed phase 29 (this result is analyzed in Section 4). These polyaromatic compounds can be precursors of creation of carbon-containing particles directly in liquid hydrocarbons.

**Figure 7:**
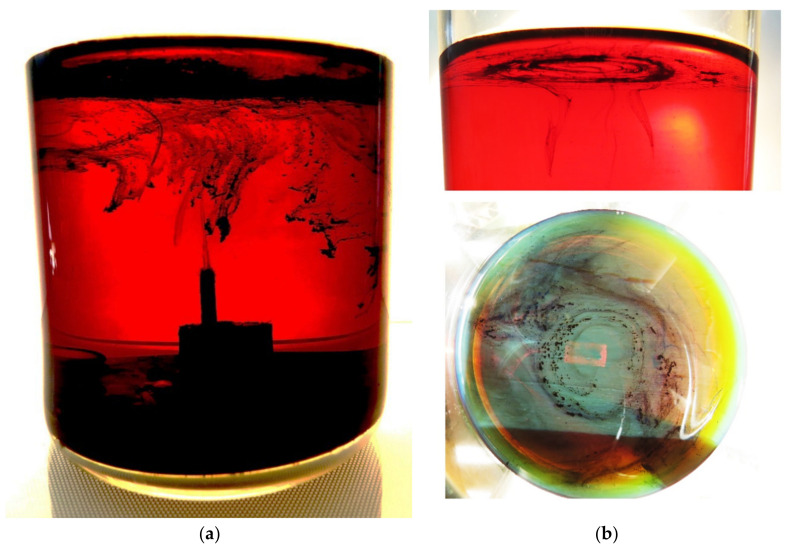
Snapshot of traces of carbonaceous particles in the engine oil just after switching-off the microwave discharge (**a**); and view of vortex structure creating by solid particles (**b**).

To answer the question, we took snapshots of discharge chamber immediately after processing of heavy hydrocarbon (engine oil) in discharge when hydrocarbon has not yet been heated (Figure 7). Engine oil with high viscosity is chosen for visualization of traces of solid particles in order to suppress the turbulence of the liquid and the spreading process of particles. The snapshot (Figure 6a) exhibits a column of soot particles spreading up from the tip of the antenna into the liquid. Thus, we can conclude that carbon-containing particles are produced in the region of gas bubble with microwave discharge at the tip of the antenna. Then, the particles are transferred into the liquid. Vortex flows (Figure 6b) emerging during heating the liquid hydrocarbon spread the solid particles over the entire volume of the liquid.

Solid particles produced by in-liquid MW plasma at atmospheric pressure in a series of alkanes (C_n_H_2n + 2_, n = 7, 8, 10, 15, 16) are studied in [31]. These hydrocarbons differ in their molecular mass and boiling points (*n*-heptane: 98.42 °C; isooctane: 99.24 °C; decane: 174.1 °C; hexadecane: 286.79 °C). The alkanes were chosen for several reasons. Firstly, as with all nonpolar hydrocarbons, alkanes possess low loss tangent in the microwave range (~10^−4^). This made it possible to neglect their direct heating by microwave field. They all have nearly the same dielectric constant (~2) in the microwave frequency range. The study [31] was run in the discharge produced by a resonance antenna (Figure 2a), which was the same in all experiments with all hydrocarbons. Secondly, all alkanes are transparent in the visible range of electromagnetic spectrum, which is important for carrying out optical and spectral measurements of in-liquid plasma.

**Figure 8:**
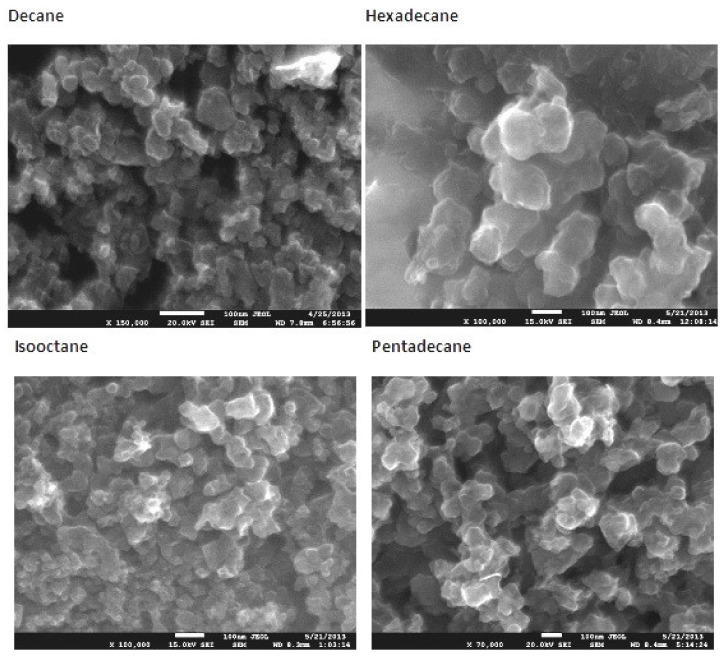
SEM micrographs of the solid products of microwave plasma in different liquid hydrocarbons.

Figure 8 shows SEM micrographs of the solid products obtained after centrifugation of various liquid hydrocarbons processed with microwave plasma. The size of the grains lays in the range of 100–200 nm, the size increasing with an increase in the number of carbon atoms per hydrocarbon molecule.

EDS analysis of the solid samples for partial contents of carbon, oxygen and copper atoms has shown that the samples consist of carbon (80–90%), oxygen (2–15%) and copper (below 2%). Oxygen content lowers and the concentrations of other elements grow with raising the number of carbon atoms in the hydrocarbon chain.

Occurrence of oxygen in the analyzed samples cannot be attributed to the action of plasma because plasma was produced in oxygen-free atmosphere. It should be noted that the carbonaceous products, after isolation from the plasma reactor, were kept in air atmosphere before subjected to further analysis by, e.g., FTIR and Raman methods. It is known that adsorption of nitrogen and oxygen on carbon materials is nearly the same, while the rate of nitrogen absorption is ten times lower than the rate of oxygen absorption. Carbon nanostructures effectively adsorb the oxygen from the ambient atmosphere [70]. Oxygen is frequently observed in the carbonaceous materials produced in plasma after exposing to the air. Insignificant amount of copper in the samples is explained by the fact that the quarter-wave antenna employed in our case to excite the discharge was made from copper. Plasma erosion of this antenna could be a source of copper contamination.

For all solid studied products, Raman spectra have exhibited a set of necessary bands permitting to categorize these solids as a “damaged graphene”, and mainly not single-layered one. NMR spectra of solid products obtained from *n*-heptane, isooctane and pentadecane reveal that generation of MW plasma in liquid alkanes leads to strong aromatization and formation of various unsaturated molecular fragments in solid products.

The results in [28], obtained in microwave discharge in liquid dodecane, show that hydrogen at a rate of 26 mL/s was simultaneously produced with carbon-contained solids at a maximum rate of 4 mg/s in this process.

**Figure 9:**
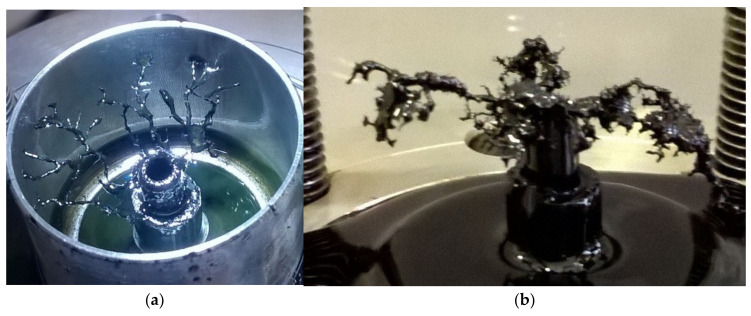
Photographs of tree-like structures formed: in microwave discharge in liquid engine oil (**a**); and in liquid residues of heavy petroleum feedstock (**b**).

Additional information should be obtained for the solid product deposited on the tip of antenna when discharge was generated in liquid hydrocarbons having high viscosity. Microwave discharge in such hydrocarbons (e.g., engine oils, crude oil and liquid residues of heavy petroleum feedstock) produces growth of tree-like structure on antenna (see Figure 9).

These solid products have special interest for the processing of crude oil and liquid residues of heavy petroleum feedstock. The results in [71,72,73] demonstrate a possible new field in application of microwave discharges in liquid hydrocarbons related with extracting a concentrate of valuable metals contained in heavy petroleum and liquid residues of heavy petroleum feedstock.

Heavy crude oil and heavy products of its processing are characterized by an increased content of compounds of some valuable metals (V, Ni, Mo, Co, etc.) [74]. These metals are usually present in combination with naphthenic acid as soaps and in the form of complex organometallic compounds such as metalloporphyrins. Existing methods for extracting metals from heavy petroleum feedstocks (deasphalting and demetallization) either do not give the required degree of purification of raw materials from metal compounds or their use on an industrial scale is not economically feasible (multi-stage, energy-consuming and capital-intensive processes). Therefore, an important task is to search for new methods for isolation of metals. The results in [71,72,73] show that microwave discharge in these liquid compounds give a promising path to solve this problem. The results are summarized in Table 3. One can see that the material deposited on the antenna contains 10–20 times higher concentrations of metals than those in the source heavy hydrocarbons.

The reason for this phenomenon is not entirely clear. It can be expected that the formation of a tree-like structure and its enrichment by metals are coupled phenomena and due to the transfer of charged particles to the antenna. It is well known that any object immersed in plasma gains negative charge relative to plasma. This causes transportation of positively charged particles from plasma to this object. Microwave antenna is such an object. The tip of the antenna is surrounded with plasma, which provides the charged particles flux to its surface. Positively charged particles are the “bricks” to build the tree-like structure. Heavy charged particles, among other things, contain cations of metal-containing oil complexes [75].

## 5. Liquid Hydrocarbons after Treatment by Microwave Discharge

**Figure 10:**
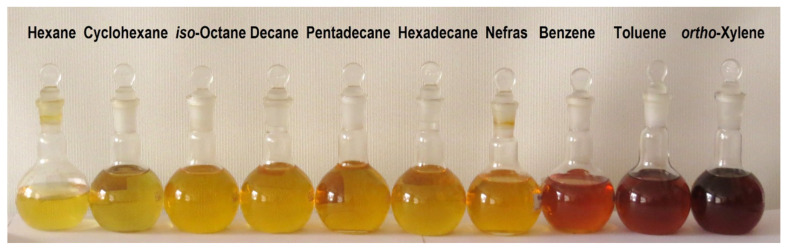
Photos of colored liquid hydrocarbons after treatment by microwave discharge and disposal of solid particles.

After removal of the carbon-containing solid particles formed in the discharge from liquid hydrocarbons (centrifuged at 3000 rpm for 10 min), the initially transparent colorless liquids acquired color (Figure 10). 

**Figure 11:**
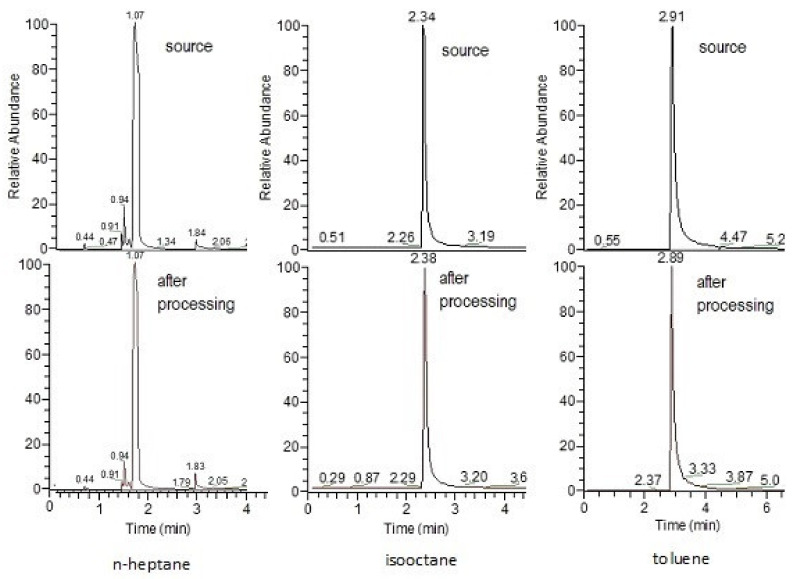
Examples of main components detected in: the source hydrocarbons (**top**); and processed hydrocarbons (**bottom**).

Hydrocarbons thus purified were analyzed by GC/MS [76]. The composition of the liquid was determined by GC/MS on a Thermo Focus DSQII gas chromatography/mass spectrometer. It was shown that the composition of the liquid hydrocarbon determined by this method does not differ from the initial one (some examples are given in Figure 11).

To measure the minor impurities that were not detected in the measurements described above, the sample was concentrated by evaporation and residue was analyzed on the same device as above. In addition, a Bruker Tensor II FT-IR spectrometer was used for the analysis.

Residues contain a wide spectrum of hydrocarbons up to C40. Detected substances such as naphthalene, acenaphthylene and dicotylphthalate have a color from light yellow to brown. They can give color to hydrocarbons after microwave plasma treatment.

FT-IR spectra of the evaporated precipitate detect the presence in the sample of unsubstituted polyaromatic compounds such as naphthalene and phenanthrene, as well as nitrogen-containing aromatic compounds such as pyroll.

In [77], a study is presented of the evaporated sediment of liquid hydrocarbons after the ignition of a microwave discharge in them with bubbling air. The authors showed that, despite the fact that plasma is created in the presence of air, oxygen- and nitrogen-containing products are practically not observed in liquid hydrocarbons. It means that the composition of the plasma has practically no effect on the surrounding hydrocarbon.

It should be noted that all these substances are contained in hydrocarbons in trace quantities.

Changes in the liquid *n*-hexane and *n*-heptane after treatment in microwave discharge are also studied in [78]. Discharge was ignited in liquids mixed with carbon fiber. Products C_8_–C_12_ were identified. In *n*-hexane, these substances are (in mg/L): phenylethyne C_8_H_6_ (0.85), styrene; 1,3,5,7-cyclooctatetraene C_8_H_8_ (0.45), ethylbenzene C_8_H_10_ (0.41), naphthalene C_10_H_8_ (0.31) and 4-propylheptane C_10_H_22_ (0.91). In *n*-htptane, they are: phenylethyne (4.9), styrene; 1,3,5,7-cyclooctatetraene (10.7), naphthalene (8.4) and biphenylene C_12_H_8_ (2.8).

It is known that the color of a liquid can be imparted by nanoparticles contained in it, which are not removed by centrifugation and do not precipitate. An example is solutions containnig fullerenes [79] or metal nanoparticles due to surface plasmon resonance [80]. To indicate the nanoparticles and determine their sizes, the method of dynamic light scattering was used with the analysis of the spectral power density of Doppler shifts (light beat spectroscopy). The measurements were carried out on a Zetatrac laser (λ = 750 nm) analyzer (NanotracWave) using the reflection method (the scattered signal recording angle was 180° with respect to the direction of the primary beam). The range of sizes of scattering particles registered by the device (their hydrodynamic diameters) was 0.001–6.500 µm. Signal processing and calculations were performed in the approximation of optically opaque spherical particles.

**Figure 12:**
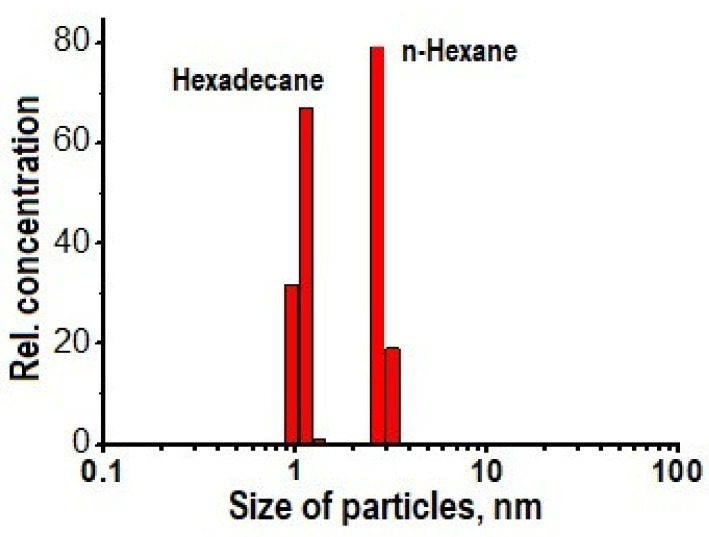
Examples of nanoparticles detected in processed hydrocarbons.

It was shown that the hydrocarbon contains nanoparticles with sizes of 1–3 nm. Some of results are shown in Figure 12.

The naphthalene and acenaphthylene found in the liquid at room temperature are crystals (melting temperature ≥80 °C), and thus it can be assumed that these crystals are the detected nanoparticles in the solution.

## 6. Basic Information about Plasma Properties (Diagnostics and Modeling)

### 6.1. Diagnostics

The main, if not the only, method for determining plasma parameters in liquids, including liquid hydrocarbons, is optical emission spectroscopy. The results of using this method for diagnosing a microwave discharge at atmospheric pressure in liquid hydrocarbons are given in [30,45,81].

**Figure 13:**
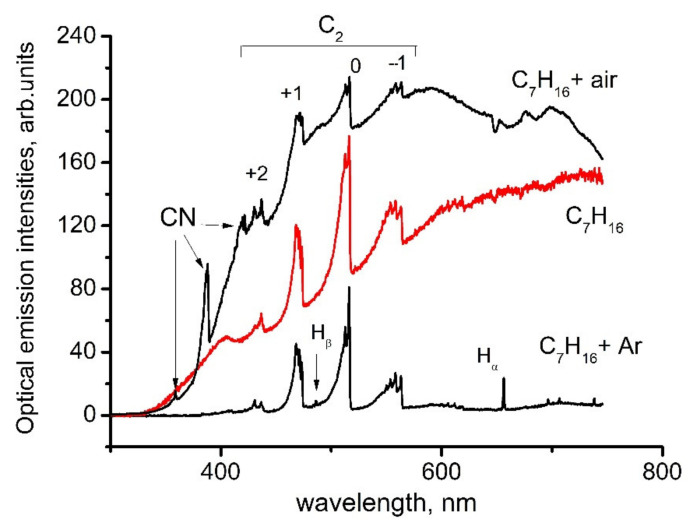
Examples of optical emission spectra of microwave discharge in liquid pure *n*-heptane and with bubbling of Ar and air. Spectra are given in arbitrary units for clear illustration the role of bubbling gases.

Optical emission spectra of microwave discharge in liquid *n*-heptane with and without bubbling of argon and air are shown in Figure 13. The spectrum of the microwave discharge without bubbling gases is represented by Swan bands (transition $C_{2}\left(d^{3}\Pi_{g}-a^{3}\Pi_{u}\right)$) sequences Δυ = 0 (maximum at 516.5 nm), Δυ = 1 (maximum at 563.5 nm), Δυ = −1 (maximum at 473.75 nm) and Δυ = −2, and the band at 436.5 nm (Δυ = −2) is overlaid with the 0-0 emission band of CH at 431.2 nm (CH transition $CH\left(A^{2}\Delta -X^{2}\Pi \right)$). No hydrogen emission lines and bands are observed. In addition, a broadband emission spectrum of solid carbon-containing particles is observed (see the curve for C_7_H_16_ in Figure 13). This continuum gives the temperature of solid particles in the range 3500–4000 K.

A similar structure of the spectrum is also observed at low argon consumption.

At high argon consumption, the structure of the spectrum changes qualitatively [43]. The H_α_ line appears in it (the threshold is 12.09 eV), and, as the argon consumption increases, the H_β_ line (the threshold is 12.75 eV) and the argon emission lines with thresholds of the order of 13.3 eV also appears. Since the excitation of the emitting states of atoms occurs in collisions with electrons, the appearance of atomic lines in the emission spectrum indicates an increase in the role of electron impact with the addition of argon. This effect is discussed in detail in [43]. In addition, the radiation intensity of the continuum decreases (see the lower curve in Figure 13), which is associated with a decrease in the rate of formation of carbon-containing particles due to dilution of hydrocarbon vapors with argon.

The appearance of hydrogen lines upon the addition of argon made it possible to determine the range of microwave field strengths in plasma. The calculations were carried out under the assumption that the excitation of the emission of hydrogen lines occurs by electron impact. The electric field strength is in the range of 2000–4000 V/cm [43].

Air bubbling leads to the appearance of CN bands (358.4, 388.3 and 421.6 nm) in the emission spectrum. The emission of hydrogen lines and bands, as in the discharge without bubbling, is not observed (Figure 13).

The unexpected results were obtained during the study of plasma emission spectra in aromatic hydrocarbons [45]. The sequence of Swan band with Δυ = 0 in spectra measured in toluene and *ortho*-xylene was overlapped with the molecular emission band with maximum at 511 nm. Attempts to measure spectra of discharge in benzene were unsuccessful as spectra were presented by continuum emission of solid particles. The most pronounced additional band was observed in experiments with toluene. With an increase in the number of CH_3_ groups in a molecule (one group is in toluene and two groups are in *ortho*-xylene), the intensity of additional band decreases and it almost disappeared in other hydrocarbons. It seems that trace of this band presents in spectra of plasma in other liquid hydrocarbons.

Analysis of literature data [45] bring to conclusion that this emission can be caused by emission of linear carbon cluster C_5_ (transition $C_{5}\left({}^{1}\Pi_{u}\to X^{1}\Sigma_{u}^{+}\right)$). This cluster was previously observed only in absorption. This means that microwave discharge in liquid aromatic hydrocarbons produced particular conditions for C_5_ generation and its emission.

Several reasons can lead to a high concentration of C_5_ in a microwave discharge in liquid aromatic hydrocarbons. First, and this fact is noted in many publications on microwave discharges in liquids, the rates of product formation in microwave discharges are higher in comparison with other discharges. This is caused by high rates of formation of active particles. Another reason of high concentration of the emitting state of the C_5_ linear cluster can be effective stabilization of hydrocarbon decomposition products, which is caused by a large temperature gradient (at the center of discharge the temperature is of 2000 K, and at the boundary the temperature is equal to the boiling point of the hydrocarbon). The observation of a new emission band only in aromatic hydrocarbons may be because the rate of formation of the solid phase in them is much higher than in other hydrocarbons. It was shown in our experiments. Therefore, it can be expected that the rate of formation of active particles is also high.

Sequences of Swan bands are often used for calculation of rotational temperature T_r_ in plasma. This procedure can be realized in two methods. One is the use of spectral devices with high wavelength resolution. It makes possible building the Boltzmann plot of rotational population to calculate the rotational temperature. Use of low-resolution optical devices give nonresolved rotational structure of spectrum and T_r_ as usual can be obtained from comparison of measured and modeled spectra of Swan sequences. The last method was used for processing of Swan sequences in [30,45,81].

Analysis of the obtained spectra of Swan bends showed that the sequence Δυ = −1 is the least noisy. Moreover, this sequence is free of overlapping with other bands. Thus, this sequence fives more reliable information on the rotational temperature. In the microwave discharge in all studied hydrocarbons, the rotational temperature T_r_ turned out to be the same and equal to 2000 ± 300 K.

In the experimental conditions described above T_r_ is equal to the gas temperature T_g_. The problem of equality of rotational and gas temperatures for these conditions was analyzed in detail in [80]. To satisfy the equality T_r_ = T_g_, it is necessary to fulfill several conditions [82]: (I) the characteristic time of energy exchange between translational and rotational degrees of freedom (τ_exch_) is much less than the time of their radiative decay (τ_rad_) and less the times of the processes leading to a change in the population of radiating levels of molecules; (II) τ_exch_ is much less than the residence time of molecules in the discharge zone; and (III) there is no radiation distortion due to reabsorption, refraction and reflections from elements of the optical system, among others. The results in [81] show that in our case these requirements are satisfied.

Thus, it was shown that the gas temperature in microwave plasma in a wide class of liquid hydrocarbons (alkanes, cyclic and aromatic hydrocarbons) at atmospheric pressure does not depend on the type of hydrocarbon and is equal to 2000 ± 300 K [45].

With decreasing pressure together with Swan bands, emission lines of atomic hydrogen appear in the emission spectrum of a microwave discharge in liquid hydrocarbons. Plasma excitation temperature determined from the relative intensities of the H_α_ and H_β_ lines was approximately 5000 K [48].

A large amount of information about plasma parameters and processes in it can be obtained by modeling a discharge.

### 6.2. Modeling

In its simplest form, modeling consists of solving electrodynamic problems that allow choosing the optimal geometry of a microwave reactor, taking into account the electrical properties of a liquid hydrocarbon, but without taking into account plasma. It gives the dimensions of the antenna and the strength of the microwave field at its end where the plasma will be generated [14,15,16,17,30,44]. For example, as noted above, this made it possible to carry out a comparative analysis of the reactor with a quarter-wavelength antenna and with coaxial energy input into the discharge and show the lower sensitivity of the latter to the antenna length (and its possible change due to the deposition of carbon-containing particles) [44].

A microwave discharge in liquid *n*-heptane at atmospheric pressure has been studied in most detail by the method of mathematical modeling. 2D, 1D and 0D models of the discharge have been developed, including those in *n*-heptane with argon bubbling.

#### 6.2.1. 2D Model: Problem Formulation and Main Results

The model describes the system with coaxial input of microwave energy in reactor filled with liquid *n*-heptane. In 2D model [19,20] the following assumptions was made:

- Gas bubbles are created by evaporation of liquid into the bubble.
- In plasma formed inside the bubble, the main ion is C_7_H_16_^+^.
- Heating of the bubble is due to Joule heating of plasma electrons.
- Cooling of the bubble is due to energy transfer to ambient liquid for evaporation and endothermic pyrolysis of *n*-heptane.
- The size and shape of the bubbles is determined by the surface tension and the sum of the pressure forces.
- Lifting of the bubble is determined by the Archimedean force and viscosity.
- The initial temperature of the liquid and gas phase is equal to the boiling point.
- A small bubble of evaporated gas of atmospheric pressure is set inside the near-electrode cavity.

Here, we note that the model contains only a simplified brutto mechanism for the dissociation of *n*-heptane, and detailed kinetics of gas products and solid carbonaceous particles is absent.

The code is based on joint solution of the Maxwell equations, Navier–Stokes equation, heat conduction equation, continuity equations for electrons (written in the ambipolar diffusion approximation) and the *n*-heptane concentration (including its thermal decomposition and dissociation by electron impact), the Cann–Hillard equation described the gas–liquid transition layer and the Boltzmann equation for free plasma electrons.

The main results of the simulations can be formulated as follows:

- Boiling process depends on the input power. There is a certain range of input power, when there is a regime of periodic formation of bubbles and their further rise.
- The plasma burns only inside the bubble, in close proximity to the central electrode. When a bubble floats up, plasma disappears inside it. This is due to the fact that the microwave field is concentrated near the end of the central electrode and falls very sharply outside it. At a power >500 W, the microwave field at the electrode end reaches 10 kV/cm. The temperature in this area is about 1500–1700 K, which is in good agreement with our experimental data. The maximum electron density is about 10^14^ cm^−3^.
- As the bubble rises, it cools very quickly due to energy transfer to ambient boiling liquid needed for its evaporation. Thus, temperature of the rising bubble very soon becomes approximately equal to the boiling point.
- For times less than 10^−3^, the dissociation of *n*-heptane occurs under the action of electron impact, and then is dominated by thermal dissociation. The role of the plasma is reduced to the formation of gas bubbles and initiation of thermal reactions.

#### 6.2.2. Modeling of Carbonaceous Particle Growth

Carbonaceous particles are one of the main products of microwave discharge in liquid hydrocarbons. According to modern views, the soot formation includes the following stages: decomposition of the starting hydrocarbon into a variety of radicals and stable molecules, the formation of molecular precursors of the soot from these fragments, surface growth and coagulation of solid particles. The growth of carbonaceous particles in microwave discharge in liquid *n*-heptane was considered in the frame of 0D [21] and 1D [22] models.

The models contain the detail mechanism for *n*-heptane pyrolysis, as published in [49,83]. Acetylene is the most important agent in the formation of soot particles. The mechanism published by Wang and Frenklach [84] for the description of pyrolysis of acetylene was used.

It is currently believed that the formation of precursors of carbonaceous particles (nuclei) comes from polyaromatic hydrocarbons (PAH) in reactions with acetylene and other hydrocarbons. According to the well-known HACA mechanism (“H-abstraction-C_2_H_2_-addition”), the growth of molecules occurs when the sequence of two stages is repeated: detachment of the H-atom from the molecule of PAH due to collision with other H-atom and addition of the molecule C_2_H_2_ from the gas phase to the vacant site.

The linear and planar mechanisms of the molecular precursor size growth were considered, but the best results in comparison with results of experiments ware obtained for planar mechanism. This mechanism is presented below.

**Figure 14:**
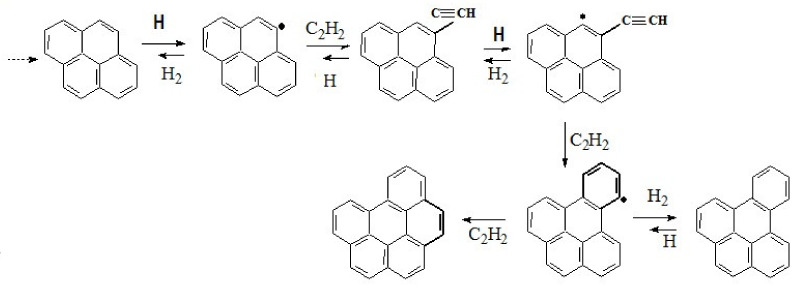
The mechanism of formation of the precursor molecule.

At the first stage of the formation of solid particles, polyaromatic hydrocarbons grow from pyrene to a nucleus molecule consisting of eight benzene rings. The mechanism of formation of the nucleus of a solid particle consists of a repeating sequence of addition of new aromatic rings in reactions with acetylene and hydrogen. Figure 14 shows the transition from pyrene to 1,12-Benzoperylene. Subsequently, these reactions are repeated until the formation of a nucleus consisting of eight benzene rings, and then the growth of a solid particle begins. Its superficial growth is carried out using the same chain of reactions. The surface of the soot particle is considered as the edge of a large PAH molecule covered with C–H bonds. This assumption determines the nature of the active centers, which interact with acetylene molecules from the gas phase, and makes it possible to calculate the growth rate of the particle surface.

For the coagulation of solid particles, an equation of the type of the Smoluchowski equation was used.

Solids are classified into 17 groups based on their size. The maximum size of the particles considered in the model is about 20 nm.

A full set of balance equations for each component of the gas phase and solid products has been solved numerically. The kinetic simulations have been implemented using COMSOL Multiphysics—Reaction Engineering Laboratory. The processes take place inside a spherical bubble, which is regarded as a continuous stirred tank reactor (CSTR) of constant pressure and volume with continuous input of gaseous *n*-heptane, which evaporates into the bubble from the external liquid.

**Figure 15:**
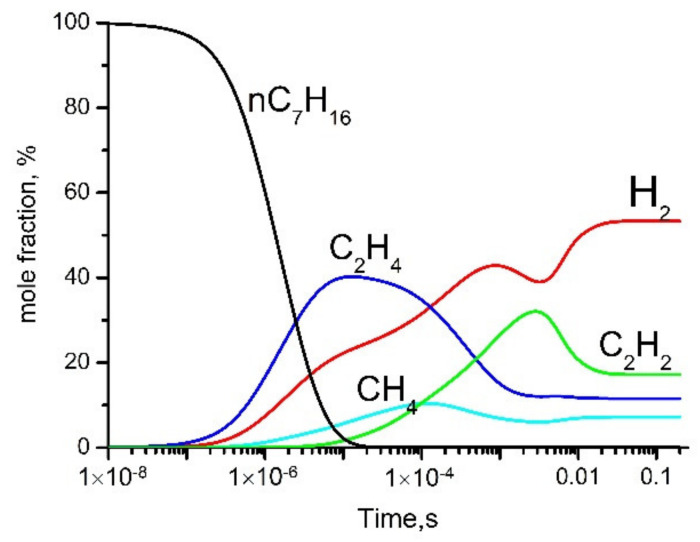
Evolution of gas phase products. Simulation at *T* = 1500 K, *p* = 1 atm.

**Figure 16:**
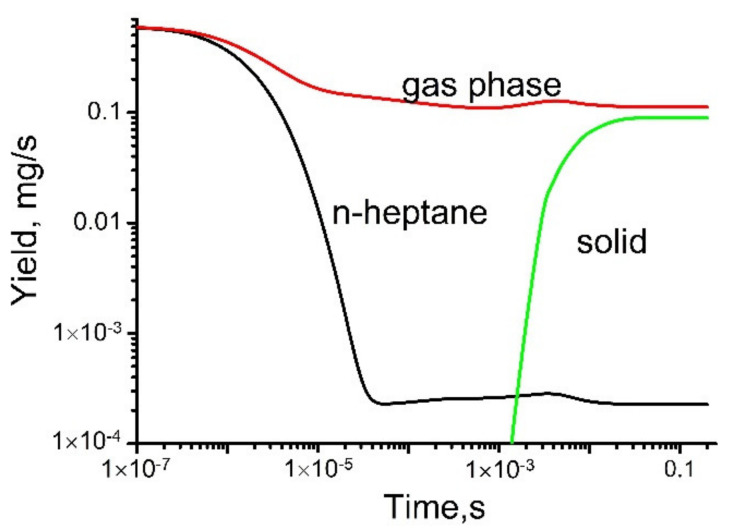
Evolution of the total yields of the gas-phase and solid products and rate of *n*-heptane loss.

Some simulation results are shown in Figure 15 and Figure 16.

The modeling approach used in [21] was applied by the authors of [22] within a one-dimensional model to study plasma processes in a gas bubble surrounded by liquid *n*-heptane. A shortened kinetic scheme of chemical processes was designed and used which gives a good match with the full scheme. The problem was solved in the approximation of spherical symmetry at atmospheric pressure. It was believed that the central electrode is located in the center of a gas bubble of a given diameter, surrounded by a liquid. The size of the bubble was taken from experiments. The model takes into account the influx of vaporized *n*-heptane molecules from the liquid phase surrounding the gas bubble.

It was shown that microwave field drops sharply near the electrode, and then is almost constant along the radius. When the gas temperature reaches about 1000 K, a sharp decomposition front of *n*-heptane appears which moves to the boundary of the gas bubble with speeds of the order of several tens of centimeters per second. The temporal distributions of the gas temperature, electron concentrations, main gas-phase products, solid particles and microwave field were obtained. Table 4 shows the comparison of calculated and obtained in experiment concentrations of main gas products. The data of modeling were obtained by averaging stationary profiles of gas products over the volume of the bubble. The calculation results are consistent with the results of experiment.

#### 6.2.3. The Role of Argon Bubbling and Charging of Carbonaceous Particle (0D Models)

Argon is often used to produce the gas bubble what simplify ignition of discharge in liquids. Thus, the discharge exists in the mixture of argon and vapor of hydrocarbon. The influence of argon on the properties of microwave plasma in bubble inside the liquid *n*-heptane is analyzed in [25] on the basis of non-stationary 0D model.

It is known that bodies immersed in plasma acquire a negative charge and are at a floating potential [85,86]. This phenomenon has been studied in detail when studying processes in the so-called “dusty plasma” [87,88]. Particle charging leads to a change in the coagulation conditions of particles and affects the process of their growth in plasma. The carbonaceous particles formed in microwave discharge in liquid *n*-heptane should be charged, and this process can influence both on plasma parameters and on particle growth. This complex problem was solved on the basis of the zero-dimensional non-stationary mathematical model described in detail in [25]. The model described in [25] was extended to take into account the charging process of particles and give possibility to analyze both effect of argon addition (with volume concentration *θ* = 0–90%) and particle charging on the plasma processes. Thus, the model [26] is the most complete for microwave discharge in plasma bubble in *n*-heptane with admixture argon as it includes equations for the kinetics of neutral and charged plasma components, equations describing the formation and growth of solid particles from *n*-heptane decomposition products, an equation for the plasma microwave field and an equation of solid particle charging.

Herem we give the main results only.

Particle charging and the addition of argon do not affect the composition of the main gas-phase products, which are hydrogen and acetylene. It does not affect the composition of positive ions. The main ion is the C_2_H_2_^+^ ion. 

**Figure 17:**
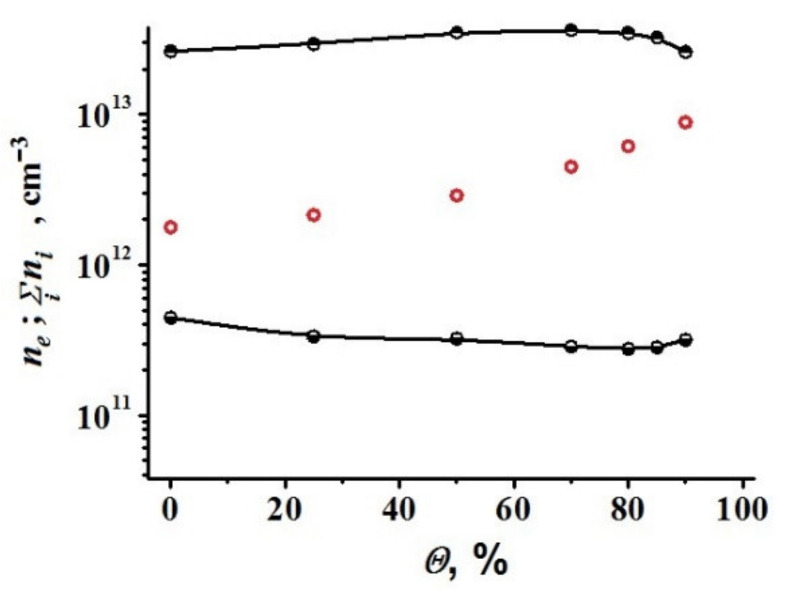
Dependences of the stationary values of the total concentration of ions (upper curve) and electrons (lower curve) on the volume fraction of argon introduced into the discharge. The curves are calculated taking into account the charge of solid particles. Hollow circles denote the equilibrium concentrations $n_{e}=\sum n_{i}$ obtained in calculations for the same power without taking into account the charge of solid particles. The power absorbed in the discharge is 50 W.

Charging the produced solid particles leads to the fact that the quasi-neutral plasma is mainly provided by a negative charge of solid particles, and the electron concentration is 1–2 orders of magnitude lower than the total concentration of positive ions. However, it should be noted that, with the introduction of a large portion of argon (80–90%), the difference between the total concentration of ions and electrons decreases (Figure 17). This is due to the fact that the total concentration of solid particles decreases in the mixture of hydrocarbon vapor diluted with argon, which reduces the effect of an additional channel for charged particles loss on their surface.

**Figure 18:**
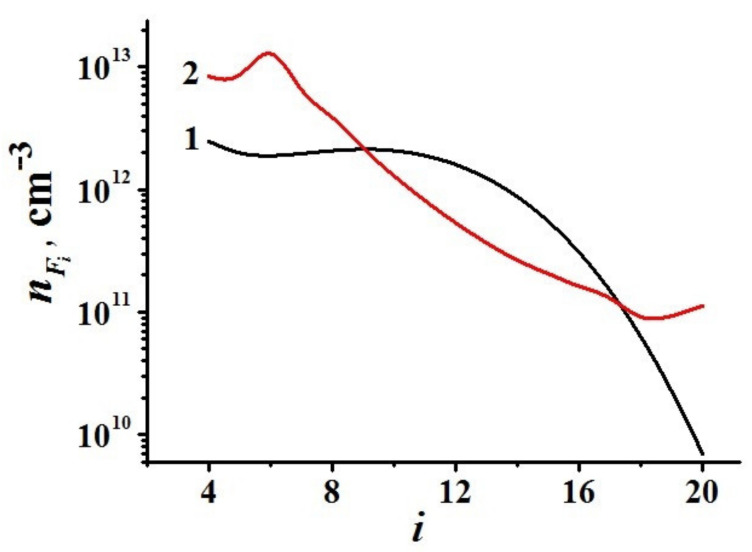
Concentrations of solid particles of groups *F_i_* depending on their size. Index *i* characterizes a solid particle containing 2*^i^* aromatic rings. Curve 1 is obtained taking into account the charging of solid particles, while Curve 2 is obtained without taking into account charging. The volume fraction of argon is *θ* = 80%. The absorbed microwave power is 100 W.

Particle charging suppresses the coagulation process for large solid particles and, accordingly, changes the size distribution of the formed solid particles and leads to the formation of maximum of the particle size distribution function in the medium-sized particle region (Figure 18).

The results obtained make it possible to determine ways to control the plasma parameters and the size of the formed solid particles.

General results presented above were obtained by simulating a microwave discharge in *n*-heptane. However, it is very likely that the general conclusions of the work on the nature of the effect of the charging of solid particles and the addition of argon will be valid for other liquid hydrocarbons.

## 7. Conclusions

Microwave discharges in liquid hydrocarbons are a relatively new and promising area in plasma physics and plasma application. Various methods have been developed for producing microwave discharges in liquids. The study of this type of discharge is still far from complete, but at present a fairly large amount of knowledge about it has been accumulated. With the help of experimental research methods and 0D, 1D and 2D modeling, a lot of information has been obtained about plasma parameters and products obtained as a result of plasma chemical reactions. Thus, the main gas products are hydrogen, acetylene, methane and ethylene, and up to 80 vol. % of them is hydrogen. Carbon-containing nanoparticles are formed as a result of plasma chemical reactions, and graphene structures are found in them. The possibility of using such discharges for the extraction of various metals from heavy oils and products of their processing is shown. It is shown that the charge of solid particles formed in the discharge has a great influence on the plasma parameters and processes in it. The unique properties of the discharge made it possible to observe in the emission spectra of the discharge in aromatic hydrocarbons a molecular band with maxima at about 511 nm, which can be attributed to the emission of the carbon complex C_5_. This molecule has not been previously observed in radiation. The liquid hydrocarbon itself remains practically unchanged after the discharge is created in it.

Many problems of microwave discharges remain unexplored. These are questions of the development of the discharge, its temporal characteristics, detailed study of physical parameters of discharges and thermodynamic state of plasma, as well as discharges with bubbling of chemically active gases. The latter systems may be of interest for the development of plasma-chemical processes, in particular, to obtain hydrogen.

## 8. Patents

Lebedev, Y.A.; Khadzhiev, S.N.; Kadiev, K.M.; Averin, K.A.; Visaliev, M.Y.; Mokochunina, T.V. Method of allocating concentrate of valuable metals contained in heavy oils and products of their processing. 2017, RU Patent 2631427.

## Figures and Tables

**Table 1:** Comparison of distributions of gas-phase products in pyrolysis of *n*-heptane [57,58,61,62] and microwave discharge in liquid *n*-heptane [43]. All values are given in wt. % (all data were recalculated on the basis of data from the original articles).

**Reference**	[61]	[57]	[59]	[58]	[62]	[43]
**Diluent**	Steam		Argon	Nitrogen	Steam	
**Temperature and pressure**	760 °C, 101 kPa	700 °C, 9.2 kPa	640 °C, 101 kPa	810 °C, 400 kPa	760 °C, 101 kPa	~1700 °C, 101 kPa
**Conversion, %**	52.51	12	15	87.8	64.3	
**Component**						
**hydrogen**	2.08	0.11	0.059	1.6	0.34	7.13
**methane**	5.39	0.91	1.11	7.1	5.41	4.02
**ethane**	1.24	0.62	1.69	2.1	1.52	0.489
**ethylene**	23.1	4.25	4.02	47.8	32.0	12.45
**acetylene**				0.6		28.39
**propane**	0.39	0.053	0.13	0.5	0.38	0.052
**propylene**	7.47	2.24	2.98	17.3	13.3	0.779
**methylacetylene**				0.3	0.00	0.247
**allene**				0.1	0.00	0.067
***n*-butane**	0.070		0.12	tr.	0.08	0.0031
**isobutane**				0.2	0.00	0.000
**1-butene**	4.84	1.83	2.26	4.6	6.28	0.105
***trans*-2-butene**	0.11			0.5	0.26	0.0024
***cis*-2-butene**	0.10					0.0019
**isobutene**				0.1	0.00	0.0010
**1,3-butadiene**	1.17		0.13	4.3	2.02	0.106
**vinylacetylene**						0.207
**1-butyne (ethylacetylene)**						0.0068
**2-butyne (dimethylacetylene)**						0.0007
**diacetylene**						0.354
***n*-pentane**						0.0074
**isopentane**	0.042				0.05	0.0038
**neopentane**						0.000
**cyclopentane**						0.0018
**1-pentene**	1.91	1.27	1.69		1.72	0.0193
***trans*-2-pentene**	0.070				0.09	0.0006
***cis*-2-pentene**	0.10					0.0004
**2-methyl-1-butene**						0.0002
**2-methyl-2-butene**						0.0012
**3-methyl-1-butene**						0.0007
**cyclopentene**						0.000
**isoprene**						0.000
**cyclopentadiene**				0.3		0.012
***n*-hexane**						0.0017
**cyclohexane**						
**methylcyclopentane**						
**cyclohexene**						0.0003
**1-methylcyclopentene**						0.00
**1-hexene**	0.82	0.71	0.81		0.55	0.00
***trans*-2-hexene**						0.0001
***cis*-2-hexene**						
**benzene**				0.4		0.020
**other NA C_5_–C_6_**				3.7		
***n*-heptane**	47.5	88.0	85.0	12.2	35.7	45.1
**toluene**				0.1		0.0005
**ethylbenzene**				traces		
**xylenes**				traces		
**styrene**				traces		
**naphthalene**				traces		
**other C_7_–C_12_**				traces		0.390
**C_12+_**				traces		
**CO**	3.57				0.24	
**CO_2_**					0.06	
**SUMMA**	100.0	100.0	100.0	100.0	100.0	100.0

**Table 2:** Relative volume concentrations (relative to hydrogen concentration) of the main gas products of microwave discharges in liquid hydrocarbons at atmospheric pressure (numbers in brackets show the volume concentration of hydrogen).

Hydrocarbon, Boiling Temperature and Structure of Molecule	H_2_	CH_4_	C_2_H_4_	C_2_H_2_	Ref.
*n*-Heptane C_7_H_16_ (T_boil_ = 98.2 °C) 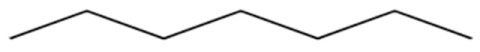	(71.2) 1	0.042	0.11	0.25	[44]
*n*-Heptane + Ar					[43]
0	1	0.059	0.18	0.27	
6.8 L/h	1	0.049	0.23	0.31	
17.3	1	0.037	0.26	0.34	
36.8	1	0.040	0.18	0.28	
*n*-Octane C_8_H_18_ (T_boil_ = 125.52 °C) 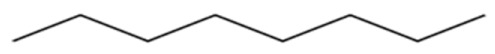	(72) 1	0.03	0.104	0.25	[44]
Decane C_10_H_22_ ((T_boil_ = 174.1 °C) 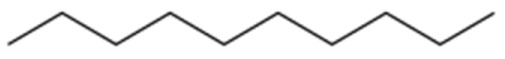	(71.7) 1	0.024	0.088	0.28	[44]
Dodecane C_12_H_26_ (T_boil_ = 216.2 °C) 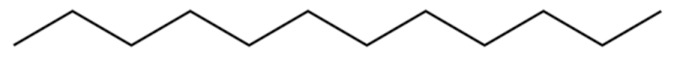	(74) 1	0.027	0.027	0.27	[28]
Pentadecane C_15_H_32_ (T_boil_ = 270.6 °C) 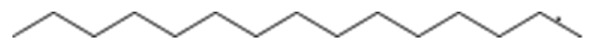	(67.6) 1	0.019	0.09	0.37	[44]
Hexadecane C_16_H_34_ (T_boil_ = 286.79 °C) 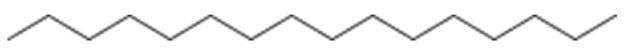	(65.6) 1	0.015	0.083	0.43	[44]
Isooctane C_8_H_18_ (T_boil_ = 99.3 °C) 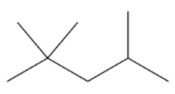	(71) 1	0.087	0.057	0.26	[44]
Cyclohexane C_6_H_12_ (T_boil_ = 80.74 °C) 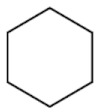	(73.4) 1	0	0.12	0.24	[44]
Benzene C_6_H_6_ (T_boil_ = 80.1 °C) 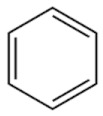	(88.80) 1	0	0	0.13	[44]
Toluene C_6_H_5_-CH_3_ (T_boil_ = 110.6 °C) 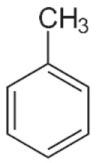	(86.1) 1	0.021	0	0.14	[44]
*Ortho*-xylole C_6_H_5_-(CH_3_)_2_ (T_boil_ = 144 °C) 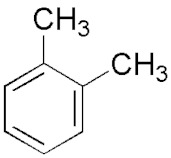	(74.6) 1	0.048	0.075	0.19	[44]
Petroleum solvent “Nefras S2 80/120” (mixture of light hydrocarbons with boiling temperature range of 33–205 °C)	(66.5) 1	0.09	0.13	0.3	[44]

**Table 3:** Illustration of enrichment of products of microwave discharge in crude oil and liquid residues of heavy petroleum feedstock in valuable metals.

Metals	Content of Metals (Weight %)			
	Source Crude Oil [72]	Tree-Like Structure on Antenna [72]	Source Heavy Petroleum Feedstock [71]	Tree-Like Structure on Antenna [71]
Al	0.00028	0.0028	0.0019	0.0068
Co	0.000053	0.00012	0.000047	0.0006
Cu	0.0002	0.0014	0.00065	0.0025
Fe	0.00064	0.0032	0.0019	0.034
Mo	0.00032	0.00067	0.067	0.98
Ni	0.008	0.013	0.0049	0.018
V	0.057	0.089	0.019	0.071
Zn	≤0.000002	0.00028	<0.000002	0.002

**Table 4:** Contents of stationary concentration of main gas products of microwave discharge in liquid *n*-heptane obtained in experiment [42] and modeling [22].

Products	H_2_ (vol. %)	C_2_H_2_ (vol. %)	C_2_H_4_ (vol. %)	CH_4_ (vol. %)
Exper. [42]/Model. [22]	71/63.5	18/23.8	8/7.5	3/5.2

## Data Availability

The data presented in this study are available on request from the corresponding author.
